# Auditing of Monitoring and Respiratory Support Equipment in a Level III-C Neonatal Intensive Care Unit

**DOI:** 10.1155/2015/719497

**Published:** 2015-10-19

**Authors:** Elena Bergon-Sendin, Carmen Perez-Grande, David Lora-Pablos, Javier De la Cruz Bertolo, María Teresa Moral-Pumarega, Gerardo Bustos-Lozano, Carmen Rosa Pallas-Alonso

**Affiliations:** Department of Neonatology, 12 de Octubre University Hospital, Biomedical Research Institute i+12, Madrid, Spain

## Abstract

*Background*. Random safety audits (RSAs) are a safety tool but have not been widely used in hospitals. *Objectives*. To determine the frequency of proper use of equipment safety mechanisms in relation to monitoring and mechanical ventilation by performing RSAs. The study also determined whether factors related to the patient, time period, or characteristics of the area of admission influenced how the device safety systems were used. *Methods*. A prospective observational study was conducted in a level III-C Neonatal Intensive Care Unit (NICU) during 2012. 87 days were randomly selected. Appropriate overall use was defined when all evaluated variables were correctly programmed in the audited device. *Results*. A total of 383 monitor and ventilator audits were performed. The Kappa coefficient of interobserver agreement was 0.93. The rate of appropriate overall use of the monitors and respiratory support equipment was 33.68%. Significant differences were found with improved usage during weekends, OR 1.85 (1.12–3.06, *p* = 0.01), and during the late shift (3 pm to 10 pm), OR 1.59 (1.03–2.4, *p* = 0.03). *Conclusions*. Equipment safety systems of monitors and ventilators are not properly used. To improve patient safety, we should identify which alarms are really needed and where the difficulties lie for the correct alarm programming.

## 1. Introduction

Random safety audits (RSAs) are a safety tool widely used in industry due to their ability to identify errors and potentially hazardous situations, thereby enabling prevention of adverse events. RSAs consist of continuously monitoring high-risk procedures in order to identify and address error-prone points in the system, difficult to detect with other methods, before they cause patient harm [1]. The application of this method in a hospital setting could be extremely valuable as it evaluates real-time clinical practice and provides immediate feedback to the staff in the unit [2]. Moreover, this method, which is applied by clinical staff, only requires simple training and involves a low cost of implementation. Despite all these advantages, RSAs have rarely been applied in the hospital setting and there is very little published data on their use as a tool for safety and quality control [3, 4].

Neonatal Intensive Care Units (NICU) have a large variety of different technological devices, most of which have built-in safety systems that help ensure appropriate use. In the field of neonatology, much attention has been paid to other types of error but less to the potential for technology-related errors, despite the fact that good patient outcomes largely depend on how the technology is used.

Consequently, we decided to implement RSAs as a safety tool to analyse the use of technology in our NICU. The primary aim of this study was to determine the frequency of proper use of equipment safety mechanisms and other measures involved in patient safety in relation to monitoring and invasive and noninvasive mechanical ventilation by performing RSAs. The study also determined whether factors related to the patient, time period, or characteristics of the area of admission influenced how the device safety systems were used.

## 2. Materials and Methods

A prospective observational study was conducted from January 1 to December 31, 2012, in a level III-C NICU with 1000 admissions annually. 120 patients with birth weight less than 1500 g per year were admitted to the NICU with a survival rate of 90%, bronchopulmonary dysplasia (BPD) rate at 36 weeks of 16.4%, and a rate for retinopathy of prematurity (ROP) grade 3 or higher of 1.4%. Our NICU is divided into three areas for critical care: a large one with 10 beds (NICU-A) and two small ones (NICU-B and NICU-C), one with 4 and the other with 5 beds, and two additional areas with 24 medium care cots. Physicians and nurses were surveyed according to the modified Delphi technique [5] on the technological devices and procedures in which the recommendations for use were apparently often not met, ensuring that the most relevant equipment and procedures were included. A total of 23 technological devices and procedures were selected (shown in Table 1) and 23 cards were produced, each containing the variables to be evaluated for each device or procedure.

During the study period, 2 days a week were randomly selected and included week days, weekends, and holidays. The shifts, early, from 8 am to 3 pm, or late, from 3 pm to 10 pm, were also randomly selected. Each study day, 2 cards were randomly selected for audit. On one day, 2 kinds of technological devices, 2 kinds of procedures, or 1 kind of device and 1 kind procedure would be audited. As it was used less frequently, the high-frequency oscillatory ventilator was audited every other day whenever it was in use. Each day of the research, a researcher (EBS or MCPG) identified selected devices or procedures in use by patients which had to be audited in order to check all study variables. The NICU staff did not know the purpose of the audit but if an error was detected which might involve a potential danger to the patient, the caregivers were immediately informed. The degree of reproducibility of the 2 investigators was analysed by simultaneous rounds of audits. In this study, the results presented are for the monitoring and respiratory support equipment, so only the audit procedure for this specific equipment is described.

### 2.1. Monitoring Equipment and Equipment for Invasive and Noninvasive Mechanical Ventilation

Information about Masimo RADICAL-7 pulse oximeters and Philips IntelliVue MP70 multimeasurement monitors was collected (see Table 2). Consideration was given as to whether the monitored patients had some type of assisted ventilation and supplemental oxygen. The outcome variable “appropriate overall use” was defined to describe when all the variables were correctly programmed in the audited monitor. Information about Dräger Babylog 8000plus conventional mechanical ventilator, Carefusion SensorMedics high-frequency oscillatory ventilator, and Viasys Infant Flow system continuous positive airway pressure (CPAP) was collected (see Table 2). The outcome variable “appropriate overall use” was defined to describe when all the variables were correctly programmed in the audited ventilator. A critical incident was defined as any situation detected concerning the devices reviewed that could put the patient in immediate risk.

### 2.2. Other Variables

Information was collected on the patient, the time, and characteristics of the NICU area where the patient was admitted in order to evaluate whether or not they influenced the use of the safety systems of these devices: birth weight; gestational age; sex; occupancy of the NICU at the time of the audit; and location of the patient.

### 2.3. Work Organization in the NICU

In the NICU, the ratio patient/nurse is 2.1 during all the shifts. The nurses are registered nurses with university education and neonatal experience. Paediatric nurse specialists were introduced 3 years ago in Spain. In relation to the doctors, 7 neonatologist and 7 paediatrics residents are in charge of the 19 intensive care beds and 24 medium care beds. During the duty (from 3 pm to 8 am) 2 neonatologist consultants and 1 resident are in charge of the neonatal unit including attention to delivery room. The nurses are responsible for programming monitor and pulse oximeter alarms following the unit's protocols or medical instructions, for controlling the water and heater temperatures, and for changing respirator and CPAP tubing. The respirator and CPAP parameters and alarms are programmed by doctors.

### 2.4. Ethical Issues

This study involved quality strategies for improving patient security and thus did not require institutional review board approval. The study consent was obtained from the head doctor and the head nurses of the unit.

### 2.5. Analysis Plan

Continuous variables are presented as mean ± SD and categorical variables as absolute and relative frequencies. The reproducibility of the observations made by the two study investigators was estimated with the kappa coefficient. The statistical significance of the comparison of proportions was determined using chi-square or Fisher's exact test from contingency tables. Comparisons of the distributions of ordinal and continuous measurements were made using the Wilcoxon-Mann-Whitney test or Student's* t*-test, as appropriate. Multivariable logistic regression analysis was used to estimate the strength of association between appropriate overall use and several covariables such as gestational age, birth weight, sex, location in the NICU, normal working day or nonworking day, shift, month, and occupancy. Results are presented as odds ratios and 95% confidence intervals (CI).

## 3. Results

**Figure 1:**
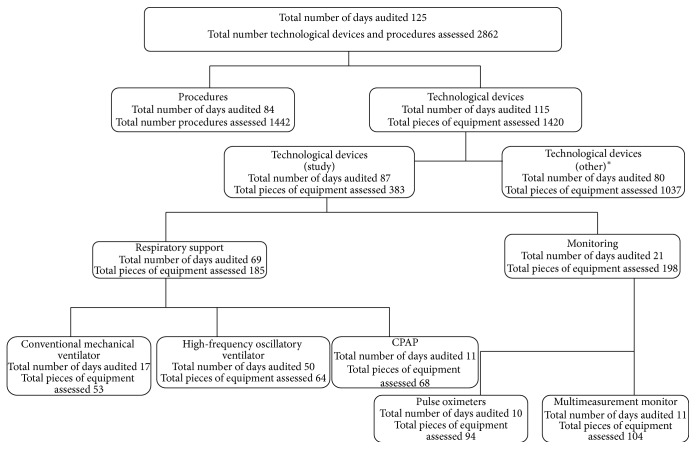
Flowchart of the audits focusing on the assessment of equipment and procedures during the study period. This study is part of a broader investigation that included other technological devices which are shown in Table 1.

During the study period, a total of 383 assessments were made of the monitoring and respiratory support equipment (see Figure 1). The overall interobserver kappa coefficient between the 2 investigators carrying out the audits was 0.93. The rate of appropriate use of the equipment safety systems and other aspects of the technology as a whole relating to patient safety was 33.68% (129/383).  Table 3 shows the rates for appropriate use of the items assessed in each device.

The variables with the worst compliance rates were in the pulse oximeters, the programming of the oxygen saturation (SpO_2_) alarms at 64.89% (61/94); in the monitors, the respiratory rate alarm at 18.18% (12/104); in the conventional mechanical ventilation respirators, the volume/minute alarm at 33.96% (18/53); in the high-frequency oscillatory ventilator, the MAP alarm at 51.56% (33/64); and in CPAP, the change of tubing at 48.53% (33/68).

Table 4 shows the results for appropriate overall use of the different pieces of equipment in relation to patient characteristics and location.  Table 5 shows the results in relation to time characteristics. Significant differences were found in the multivariate analysis, with improved overall usage during weekends and holidays, OR 1.85 (1.12–3.06, *p* = 0.01), and on the late shift, OR 1.59 (1.03–2.4, *p* = 0.03). The occupancy rate when appropriate use was found was 95.56% and 93.02% when inadequate use was detected (*p* = 0.002). There were no significant differences in any of the other variables studied.

Critical incidents were found in 9 (2.34%) of the 383 devices reviewed. These were pulse oximeter not working in 2 patients requiring monitoring; alarms not programmed in invasive blood pressure monitoring in 3 unstable patients; and no water in the heater of 4 respirators.

## 4. Discussion

This prospective observational study shows that only a third of evaluated monitors and respiratory support equipment had all the parameters well programmed. Contrary to what might be expected, weekend and holidays and late shifts, when there are fewer neonatologists in the NICU, were the periods when we found that the evaluated equipment parameters were best programmed. During the study period the NICU occupancy rate was higher than 90%. Patient characteristics and location in the unit did not influence the appropriate use of the monitors and respiratory support equipment.

To our knowledge, this is the first published study with data on real-time audits of monitoring and respiratory support equipment in a level III-C NICU. Compared to the large number of published studies on other aspects of neonatal patient safety [10–12], there are surprisingly few literature references reporting errors directly related to the equipment used [3, 13, 14]. For this reason, it has not been possible to compare many of our results with other studies. When we designed the study, we believed that our results were going to be much better than those we finally obtained after the audits.

One of the most disturbing findings in relation to the monitoring equipment was the poor protocol compliance for programming SpO_2_ alarms, results similar to those found by other authors [3, 13]. In most cases, this consisted of not programming the upper SpO_2_ alarm limit in patients on supplemental oxygen, which exposes the patient to a greater risk of hyperoxia and the widely described adverse effects on lung, retina, and brain [15–17]. There is currently a great deal of concern about what the optimal SpO_2_ ranges are for premature infants and numerous studies are being conducted in this area [16–19]. However, all the efforts will be of little effect if, on implementing the recommendations in different NICUs, the SpO_2_ alarm limits are not properly set.

Despite the high rate of inadequate programming of SpO_2_ alarms, our BPD and grade 3 or higher ROP rates are not high. Nevertheless, this does not justify the poor compliance in programming these alarms in view of the other repercussions on the newborns in our unit.

One of the most surprising results was finding a higher percentage of appropriate overall use at the weekend and during the late shift. It is not easy to explain this result. At the weekend and during late shifts, there are only two neonatologists on duty. The number of nurses is always the same on all shifts. Programmed procedures are usually not performed. There are, therefore, fewer interruptions, allowing a greater degree of concentration on the part of the staff. The nurses probably work better in the quieter, calmer atmosphere on the weekend. These aspects need to be confirmed since it does not seem logical that the use of technology should be better when there are fewer doctors working.

Also remarkable is that appropriate use of the equipment is better when there is higher occupancy. A tendency towards better use would be expected with lower occupancy and not the other way round. In our NICU, the occupancy rate is always very high and although the difference is statistically significant, it may not be clinically relevant.

We also wanted to find out whether the use of technology was different in the months of July/August/September compared to the rest of the year, as this is considered a high-risk period for adverse events due to the fact that, in Spain, it is a vacation period during which less-experienced nurses are recruited. For example, our group has reported a higher rate of unplanned extubation over the summer months [20]. However, no differences were found in the use of technology.

With regard to adverse events, higher rates have been reported in the most premature patients and those weighing less than 1500 g [21]. In our study, these patient characteristics had no influence on the use of the equipment safety systems or on other safety-related aspects of the technology evaluated. The incidents found in 2.34% of the devices reviewed were considered critical for the serious consequences they could have had for the patient. This rate is not insignificant and NICUs are highly complex units, in which high-risk situations continually occur. In all the above cases, the patient's care provider was immediately informed to resolve the problem.

A major problem in the NICUs is the large number of alarms used which produce a high level of noise. There are reports that exposure to high levels of noise in newborns, particularly when premature, can have potentially negative effects on their stability and their future neurodevelopment [22, 23]. Moreover, there are studies which show that 72–99% of the alarms that go off are not actually related to any real patient event [24]. This can lead to a situation where NICU staff suffer from “clinical alarm fatigue,” the term used to describe the phenomenon of medical providers becoming too desensitized due to the constant noise emitted from monitors [25, 26]. It could also lead to staff becoming less rigorous and not programming alarms which they believe often sound without signifying a real clinical problem for the patient. In light of the above, there is an attempt nowadays to design “smart alarms” which manage information in an integrated manner using algorithms, thereby improving the sensitivity and specificity of the alerts [27].

One limitation to our study was that although the staffs were unaware of the objectives of the observation they were, however, aware of being watched and this may have influenced their usual practices. Nevertheless, we do not believe that this aspect influenced the results. When the different quarters of the year were compared, no changes were found in staff behaviour over the course of the study period. Another limitation is that the night shift was not included in the observations. Perhaps our results cannot be generalized because the organization of other units and the degree of professional training may differ between countries and units, but these results may encourage other units to audit the technology they use in an effort to identify areas for improvement.

The results of this study have been disseminated to the NICU staff and different strategies have been introduced to improve the appropriate use of equipment. Probably, as in other aspects concerning safety, checklists are useful to evaluate the appropriate settings of monitors and respirators. The companies that design monitoring and respiratory support systems are trying to incorporate an increasing number of alerts to ensure proper use of the equipment and to improve the safety of the newborn. However, our study shows that a large number of these safety systems are not properly used and do not benefit the patient. To improve patient safety, we should seek to identify which alarms are really needed and where the difficulties lie for the correct alarm programming in the monitors and support respiratory equipment.

## Figures and Tables

**Table 1:** All the technological devices and procedures audited in the research. In bold font are the technological devices included in the present study.

Technological devices	Procedures

**Masimo RADICAL-7 pulse oximeter**	Safety
**Philips Intellivue MP70 multimeasurement monitor**	Hand hygiene
**Dräger Babylog 8000plus ventilator**	Prescription of medication
**SensorMedics Carefusionhigh-frequency oscillatory ventilator**	Administration of medication
**Viasys Infant Flow system CPAP**	Administration of enteral nutrition
Dräger Caleo and Ohmeda Giraffe incubators	Administration of fluid therapy
Alaris medication pumps	Developmental centered care
Alaris fluid therapy pumps	Identification and treatment of pain
Alaris enteral nutrition pumps	Patient identification
Phototherapy lamps	Written information for parents
Ikaria Nitric Oxide equipment	Handling of the lines
Inspiration Healthcare Tecotherm hypothermia equipment	

**Table 2:** Definitions of appropriate programming of the variables assessed for the technological devices. ECG: electrocardiogram; PIP: peak inflating pressure; MAP: mean airway pressure; CPAP: continuous positive airway pressure; PEEP: positive end-expiratory pressure.

Appropriate programming		
Pulse oximeter	SpO_2_ alarms	Lower limit 85% and upper limit 95% (as per protocol) or as instructed by a doctor
	Heart rate alarms	Lower limit 95 and upper limit 195 bpm or as instructed by a doctor
	Alarm volume	50–70 dB
	Sensor status	Clearly defined curves without artefacts

Multimeasurement monitor	SpO_2_ alarms	Lower limit 85% and upper limit 95% (as per protocol) or as instructed by a doctor
	Heart rate alarms	Lower limit 95 and upper limit 195 bpm or as instructed by a doctor [6]
	Respiratory rate alarms	Lower limit 30 and upper limit 70 rpm or as instructed by a doctor [6]
	Blood pressure alarms	±20% of the normal value for the patient's gestational age [6]
	Alarm volume	50–70 dB
	Sensor status and ECG pads	Clearly defined curves without artefacts

Conventional mechanical ventilator	Volume/minute alarm	±20% of the normal volume/minute for a newborn [7, 8]
	Apnoea alarm	20 seconds
	Alarm volume	50–70 dB
	Peak limit in volume guarantee mode	At least 5 to 10 cm H_2_O above the working PIP [9]
	Respirator flow	6-7 lpm for number 2.5 endotracheal tubes
		8–10 lpm for number 3 and number 3.5 endotracheal tubes
	Position of the flow sensor	Slightly above the level of the patient
	Heater	With water and temperature set at 37°C
	Change of tubing	As per unit policy (<7 days)

High-frequency oscillatory ventilator	MAP alarms	±3 points of the programmed MAP
	Position of tubing	Running straight and at the height of the patient
	Heater	With water and temperature set at 37°C

CPAP	PEEP	As prescribed
	Heater	With water and temperature set at 39°C
	Change of tubing	As per unit policy (<7 days)

**Table 3:** Rates of appropriate use for the variables evaluated in each device and technology as a whole. SpO_2_: oxygen saturation; VG: volume guarantee ventilation; MAP: mean airway pressure; CPAP: continuous positive airway pressure; PEEP: positive end-expiratory pressure.

Technological devices (*N* assessments)	Appropriate use (*N*)	Appropriate use % (confidence interval)
Pulse oximeters (*N* 94)		
SpO_2_ alarms	61	64.89 (55.24–74.54)
Heart rate alarms	87	92.55 (87.25–97.86)
Alarm volume	94	100 (96.15–100)
Sensor status	94	100 (96.15–100)
Appropriate overall use	58	61.70 (51.88–71.53)
Monitors (*N* 104)		
SpO_2_ alarms (*N* 104)	58	55.77 (46.22–65.31)
Heart rate alarms (*N* 104)	84	80.77 (73.19–88.34)
Respiratory rate alarms (*N* 66)	12	18.18 (8.87–27.49)
Blood pressure alarms (*N* 64)	30	46.88 (34.49–59.10)
Alarm volume (*N* 104)	104	100 (96.51–100)
State of ECG pads (*N* 75)	68	90.67 (84.08–97.25)
Appropriate overall use (*N* 104)	22	21.15 (13.30–29.00)
Conventional mechanical ventilator (*N* 53)		
Volume/minute alarms	18	33.96 (21.21–46.71)
Apnoea alarm	48	90.57 (82.69–98.44)
Peak pressure limit in VG (*N* 42)	38	90.48 (81.59–99.35)
Flow appropriate to the endotracheal tube size	48	90.57 (82.69–98.44)
Adequate position of the flow sensor	33	62.26 (49.21–75.31)
Change of tubing	26	49.06 (35.59–62.52)
Alarm volume	53	100 (93.27–100)
Heater temperature	53	100 (93.27–100)
Water in the heater	52	98.11 (94.45–100)
Appropriate overall use	6	11.32 (2.79–19.85)
High-frequency oscillatory ventilator (*N* 64)		
MAP alarms	33	51.56 (39.32–63.81)
Position of the tubing	46	71.88 (60.86–82.89)
Heater temperature	63	98.44 (95.40–100)
Water in the heater	61	95.31 (90.13–100)
Appropriate overall use	24	37.50 (22.64–49.36)
CPAP (*N* 68)		
PEEP	63	92.65 (86.44–98.85)
Change of tubing	33	48.53 (36.65–60.41)
Heater temperature	42	61.76 (50.21–73.32)
Water in the heater	68	100 (94.72–100)
Appropriate overall use	19	27.94 (17.28–38.61)
Technology as a whole (*N* 383)	129	33.68 (28.81–38.54)

**Table 4:** Rates for appropriate overall use and variable with lower rates of appropriate programming of the different pieces of equipment in relation to patient characteristics and location in the Neonatal Intensive Care Unit. NICU: Neonatal Intensive Care Unit; SpO_2_: oxygen saturation; MAP: mean airway pressure; CPAP: continuous positive airway pressure.

Technological resources (*N* assessments)	Birth weight (g) (*N* assessments)			Gestational age (weeks) (*N* assessments)			Sex (*N* assessments)			Location (*N* assessments)		
	<1500	≥1500	*p*	<32	≥32	*p*	Male	Female	*p*	NICU-A	NICU-B + C	*p*
Pulse oximeter (*N* 94)												
% appropriate overall use (*N*)	66 (33)	55.81 (24)	0.31	64.29 (36)	57.89 (22)	0.53	60.47 (26)	62.75 (32)	0.82	57.78 (26)	65.31 (32)	0.45
Multimeasurement monitor (*N* 104)												
% appropriate overall use (*N*)	24 (12)	18.52 (10)	0.49	26.32 (15)	14.89 (7)	0.15	26.53 (13)	16.36 (9)	0.20	18.33 (11)	25 (11)	0.41
Conventional mechanical ventilator (*N* 53)												
% appropriate overall use (*N*)	0.09 (2)	13.79 (4)	0.60	10 (3)	13.64 (3)	0.68	10 (3)	13.04 (3)	0.72	13.89 (5)	5.88 (1)	0.39
High-frequency oscillatory ventilator (*N* 64)												
% appropriate overall use (*N*)	32.5 (13)	45.83 (11)	0.28	31.82 (14)	50 (10)	0.16	32.43 (12)	44.44 (12)	0.32	54.17 (13)	27.5 (11)	0.03
CPAP (*N* 68)												
% appropriate overall use (*N*)	28.89 (13)	27.27 (6)	0.89	29.63 (16)	21.43 (3)	0.54	22.86 (8)	33.33 (11)	0.33	22.73 (5)	30.43 (14)	0.50
Technology as a whole (383)												
% appropriate overall use (*N*)	35.27 (73)	31.98 (55)	0.50	34.85 (84)	31.91 (45)	0.55	31.96 (62)	35.45 (67)	0.46	32.09 (60)	35.20 (69)	0.51

**Table 5:** Rates for appropriate overall use and variable with lower rates of appropriate programming of the different pieces of equipment in relation to time characteristics. SpO_2_: oxygen saturation; MAP: mean airway pressure; Vol/min alarms: volume/minute alarms; CPAP: continuous positive airway pressure.

Technological resources (*N* assessments)	Day			Shift			Quarter		
	Normal working day	Nonworking day	*p*	Early	Late	*p*	3rd quarter	Rest of year	*p*
Pulse oximeter (*N* 94)									
% appropriate overall use (*N*)	64.56 (51)	46.67 (7)	0.19	65.12 (28)	58.82 (30)	0.53	76.92 (20)	55.88 (38)	0.06
Multimeasurement monitor (*N* 104)									
% appropriate overall use (*N*)	16.05 (13)	39.13 (9)	0.01	16.22 (12)	33.33 (10)	0.05	19.35 (6)	21.92 (16)	0.76
Conventional mechanical ventilator (*N* 53)									
% appropriate overall use (*N*)	8.51 (4)	33.33 (2)	0.07	10.34 (3)	12.50 (3)	0.80	18.75 (3)	8.11 (3)	0.26
High-frequency oscillatory ventilator (*N* 64)									
% appropriate overall use (*N*)	25.53 (12)	70.59 (12)	0.001	38.46 (15)	36 (9)	0.84	35 (7)	38.64 (17)	0.78
CPAP (*N* 68)									
% appropriate overall use (*N*)	27.91 (12)	28 (7)	0.99	22.86 (8)	33.33 (11)	0.33	35.71 (5)	25.93 (14)	0.46
Technology as a whole (*N* 383)									
% appropriate overall use (*N*)	30.98 (92)	43.02 (37)	0.03	30 (66)	38.65 (63)	0.07	38.32 (41)	31.88 (88)	0.23
